# Giant cell arteritis: is the clinical spectrum of the disease changing?

**DOI:** 10.1186/s12877-019-1225-9

**Published:** 2019-07-29

**Authors:** Miguel Á. González-Gay, Miguel Ortego-Jurado, Liliana Ercole, Norberto Ortego-Centeno

**Affiliations:** 1 Division of Rheumatology and Epidemiology, Genetics and Atherosclerosis Research Group on Systemic Inflammatory Diseases, Hospital Universitario Marqués de Valdecilla, Instituto de Investigación Marqués de Valdecilla, 39011 Santander, Spain; 2 University of Cantabria, Santander, Spain; 3 Cardiovascular Pathophysiology and Genomics Research Unit, School of Physiology, Faculty of Health Sciences, University of the Witwatersrand, Johannesburg, South Africa; 4 Emergency Health Services Agency-061 (EPES-061), Granada, Spain; 5 Medical Department, Roche Pharma, Madrid, Spain; 6 Autoimmune Diseases Unit, Hospital Universitario San Cecilio, Instituto de Investigación Biosanitaria de Granada (IBS. GRANADA), Department of Internal Medicine, Professor of Medicine of the University of Granada, Granada, Spain

**Keywords:** Giant-cell arteritis, Polymyalgia rheumatica, FDG-PET/CT

## Abstract

**Background:**

Giant cell arteritis is a vasculitis of large and middle-sized arteries that affects patients aged over 50 years. It can show a typical clinical picture consisting of cranial manifestations but sometimes nonspecific symptoms and large-vessel involvement prevail. Prompt diagnosis and treatment is essential to avoid irreversible damage.

**Discussion:**

There has been an increasing knowledge on the occurrence of the disease without the typical cranial symptoms and its close relationship and overlap with polymyalgia rheumatica, and this may contribute to reduce the number of underdiagnosed patients. Although temporal artery biopsy is still the gold-standard and temporal artery ultrasonography is being widely used, newer imaging techniques (FDG-PET/TAC, MRI, CT) can be of valuable help to identify giant cell arteritis, in particular in those cases with a predominance of extracranial large-vessel manifestations.

**Conclusions:**

Giant cell arteritis is a more heterogeneous condition than previously thought. Awareness of all the potential clinical manifestations and judicious use of diagnostic tests may be an aid to avoid delayed detection and consequently ominous complications.

## Background

Giant cell arteritis (GCA) is a non-necrotizing granulomatous vasculitis affecting large and middle-sized arteries. Histologic changes, mainly inflammatory infiltrates with the presence of multinucleated giant cells between the media and intima layers and disruption of the internal elastic lamina, ultimately leads to partial or complete obstruction of local arterial blood flow with the corresponding clinical manifestations of ischemia [1–6]. Branches of the aortic arch are most often affected. However, non-specific clinical features related to the general inflammatory state, such as low-grade fever, malaise, fatigue, constitutional symptoms or anemia, may prevail in some cases. In this later group of patients, a lower index of suspicion may yield a significant diagnostic and therapeutic delay with potential serious consequences [7]. In addition, involvement of the aorta is associated with a higher risk for developing aneurysms and dissection over the course of the disease, especially in the thoracic segment [8–10].

Most common local symptoms include acute headache, scalp pain, jaw, tongue or limb claudication, and visual deficits. Clinical features of polymyalgia rheumatica (PMR) may be present before, simultaneously or after diagnosis of GCA has been established [11, 12]. Other findings may occur in a minor proportion, including peripheral neuropathy, dry cough, sore throat or stroke [13]. It is remarkable that strokes affect more often the vertebrobasilar territory at early stages and the carotid branches during follow-up [14, 15]. In rare cases, complete arterial occlusion can end in infarction of distal territories such as the scalp, tongue or limbs.

Ocular involvement may lead to irreversible visual loss, one of the most feared complications, and, for this reason, early diagnosis and treatment of GCA is of major importance. It usually presents as an anterior optic ischemic neuropathy or less commonly as retinal artery occlusion. In addition, visual impairment can be the result of stroke affecting the vertebra-basilar territory [16]. Previous episodes of amaurosis fugax have been associated with an increased risk for permanent visual loss [16]. Other ocular manifestations such as episcleritis, scleritis or extraocular muscle palsies are less commonly reported [17–19].

On physical examination, it is important to look for abnormalities in temporal arteries (i.e. thickening, tenderness, beading or reduced pulsation) [20, 21], but attention must also be focused on the remaining peripheral arterial territories to disclose pulse deficits, bruits or asymmetric arterial pressure measurements in any of the four limbs [22]. Although there is no specific laboratory marker for the disease, acute phase reactants (platelets, erythrocyte sedimentation rate and/or C reactive protein) are elevated in the majority of patients [23]. Conversely, normal values are not sufficient to rule out the diagnosis when there is a strong clinical suspicion. Anemia can also be present, as well as raised liver enzymes [2, 13, 23].

Temporal artery biopsy, performed with appropriate technique, is considered the gold-standard for diagnosis [1]. Nevertheless, the most recent EULAR guidelines consider that early imaging test, such as temporal artery ultrasound, performed by a trained specialist using appropriate operational procedures and settings, may be an alternative to the temporal artery biopsy [24]. Treatment should not be delayed due to pending biopsy results, and it has been demonstrated that histologic changes are recognizable within two weeks after starting therapy [25, 26]. Advances in imaging over the last few years have constituted a major step forward in the diagnosis of GCA. Besides the use of duplex ultrasonography of the temporal artery for the diagnosis of GCA in patients presenting with the typical pattern of cranial manifestations, other Imaging techniques, such as magnetic resonance, computed tomography (CT)-angiography, and [18F]-fluorodeoxyglucose (FDG)-positron emission tomography (PET/CT) can help to diagnose patients with predominant extracranial manifestations, many of them presenting with atypical manifestations of the disease [24]. These new imaging techniques allows to identify clinically silent large vessel involvement [27]. Experts in the field consider that in some cases they may also be used to monitor response to treatment.

Glucocorticoids are the cornerstone of treatment [2–6]. Some adjuvant therapies, such as methotrexate, have been used as glucocorticoid-sparing agents, thus minimizing their adverse effects, with conflicting results in terms of safety and clinical benefit [28]. It is also recommended to use low-dose aspirin to reduce the risk of associated cardiovascular events [20, 29, 30]. More recently, open-label studies and clinical trials have shown that the use tocilizumab, a novel humanized monoclonal antibody against IL-6, yields significant efficacy in achievement of sustained, corticosteroid-independent remissions [31, 32].

The aim of this report is to help physicians to early recognize symptoms of GCA, with special emphasis on those patients with atypical or extra-cranial manifestations (“occult” GCA), in order to perform an appropriate diagnosis, since it is crucial to prevent severe complications.

## Main text

### Changing epidemiology

GCA is the most common type of vasculitis in Europe and North America. It is a disease of the elderly, with a peak of prevalence in the seventh and eighth decades of life [33]. In fact, one of the most constant diagnostic criteria has been an age over 50 years. With the progressive expansion of life expectancy over the last decades and the consequent aging of general population, along with shortened periods from disease onset to final diagnosis, the prevalence of GCA tends to increase gradually despite steady incidence rates described in some studies. GCA is usually more frequent in women, with an average ratio around 2:1, and more common in patients of Scandinavian origin [3, 5, 13, 33]. A genetic predisposition has been found, with special relevance of some alleles of the HLA class II region, in particular those with HLA-DRB*04 [34, 35].

The etiology is still unknown, but certain parallelism of peak incidences with some infectious outbreaks and detection of viral molecules in biopsy specimens have tempted some investigators to hypothesize than an infection might at least trigger the inflammatory reaction. However, the exact pathogenic explanation of this finding, if any, remains to be elucidated. Microorganisms that have been implicated include varicella zoster virus, *Mycoplasma pneumoniae*, *Chlamydia pneumoniae*, and parvovirus B19 [36–38], but infectious agents have not been definitely proven to play a major causal role nor be the sole immunologic trigger [39].

Incidence of GCA was found to increase in some regions [40], in other studies there was a trend towards lower rates [41, 42], and others described stability [43]. Higher awareness of the disease by attending physicians and underestimation of more atypical extra-cranial forms in the past may have played a role in these variations. Interestingly, probably because physicians are more familiar with the disease, some series have shown a decline of visual manifestations in recent years [18, 40, 44].

### GCA and PMR, two overlapping conditions

PMR is an inflammatory disorder that usually presents as severe pain and stiffness of shoulders and proximal arms. Involvement of other regions, mainly the neck, pelvic girdle and thighs, can also occur. Raised laboratory inflammatory markers are generally observed. Ultrasonographic findings (characteristic bursitis and tenosynovitis) have been added to the latest ACR and EULAR preliminary classification criteria [45]. PMR usually has a good and rapid response to glucocorticoid therapy at lower doses than those required for treating GCA [12].

GCA shares epidemiological, physiopathological and clinical features with PMR, and they often overlap in a synchronic or metachronic manner in the same patient [11]. In fact, for some authors they comprise two variants of a single disease. It is estimated that about 20% of patients with PMR have associated GCA [46, 47], although this frequency was higher, up to 50%, in temporal-artery-biopsy-proven cases [48]. Moreover, when imaging techniques such as FDG-PET/CT are performed in patients with PMR, they have shown that at least one third of them may have large-vessel involvement that may be clinically silent and without associated typical cranial ischemic manifestations [12]. A recent study has disclosed that large-vessel vasculitis involvement is often observed in patients with persistent PMR who have inflammatory low back pain, predominant pelvic girdle and or diffuse limb pain [49].

Therefore, it is advocated that patients with a newly diagnosis of PMR should be screened for large-vessel inflammation and concomitant GCA using clinical predictors [49] and whenever confirmatory imaging techniques were available. In addition, close observation should be maintained over the entire course of the disease, since typical features of GCA and ischemic events (including visual deficits) have been described during the follow-up of patients with an initial diagnosis of isolated PMR, thus requiring higher corticosteroid doses to avoid irreversible damages [50, 51], especially blindness [52]. “Pure” (isolated) PMR patients tend to show lower values of inflammatory parameters and milder degrees of anemia and thrombocytosis compared to those with PMR associated with clinical features of GCA [53]. Also, patients with suspected PMR who do not have a good response to low-dose glucocorticoids or show marked systemic symptoms may actually represent a form of extra-cranial GCA [12].

### Cranial vs extra-cranial disease

In recent years there have been a growing interest and knowledge on cases with involvement of extra-cranial territories, in contrast to the “classic” cranial manifestations. Incidence of extra-cranial disease seems to have been underestimated in the past. This was formerly supported by some post-mortem studies and histological analysis of case series of vascular surgery of the aorta or its main branches [54]. The widespread use of whole-body imaging techniques (i.e. FDG-PET/CT and CT or MRI angiography) and a greater awareness of the disease in patients with atypical manifestations will probably raise this incidence even more in the future.

Most patients with a recent diagnosis of temporal-artery-biopsy-proven GCA have no clinical evidence of vascular ischemic manifestations [48, 55]. However, imaging techniques often show asymptomatic extra-cranial vascular involvement in these patients and support the claim that GCA involves arteries far beyond the temporal and other cranial arteries [56, 57]. Moreover, the risk of developing aortic aneurysm or dissection has been estimated up to 17 times greater than in patients without GCA, additionally increasing when other cardiovascular risk factors such as hypertension are present [8, 10]. For these reasons, some authors advocate screening imaging of the aorta at the time of diagnosis and during follow-up, since symptoms usually develop several years later [10, 58]. The presence of cardiovascular risk factors of atherosclerosis at the time of diagnosis of GCA may influence the development of severe ischemic manifestations of the disease [59]. On the other hand, some authors consider that GCA patients with a pattern of extra-cranial vascular involvement, without the typical cranial ischemic manifestations of the disease, have lower risk of having ophthalmologic complications over the course of the disease, but in turn they are more prone to relapses and large-vessel complications [60, 61].

Isolated extra-cranial GCA represents a diagnostic challenge since signs and symptoms may be nonspecific (fever, fatigue, weight loss) unless local ischemic manifestations appear. A significant proportion of patients with ischemic features attributed to atherosclerosis or embolic origin could represent an extra-cranial form of this vasculitis, particularly when cardiovascular risk factors are scarce or absent. In addition, laboratory inflammatory markers tend to be lower in this group of patients [62]. This difficulty in diagnosis often leads to a greater diagnostic delay compared with patients suffering typical cranial symptoms [7, 63]. Interestingly, patients with predominant large-vessel-vasculitis pattern are younger and have more commonly PMR features and longer duration of symptoms at GCA diagnosis than those with predominant cranial manifestations [60, 61]. Moreover, the frequency of a positive temporal artery biopsy in these is much lower than in those with the classic pattern of GCA [60, 63]. Because of that, in these patients the diagnosis must often be relied on large-vessel inflammatory signs on imaging tests, as retrieval of arterial specimens in other territories entails significant morbidity.

### Current diagnostic tools and diagnostic challenges

Although temporal artery biopsy is considered by many clinicians as the gold-standard for the diagnosis of GCA [1], focal or segmental inflammatory changes and technical pitfalls of the procedure make it difficult to confirm this condition in a significant proportion of patients. Diagnostic yield of biopsy varies greatly depending on the length of the excised specimen and whether it is performed unilaterally or bilaterally [64, 65]. The traditional recommendation was to obtain an arterial sample of at least 2–3 cm of length, although more recent studies consider that 1 cm could be enough for histologic diagnosis.

Some authors have designed predictive models as an aid to select those patients with higher probability of having GCA, but it should be kept in mind that these calculators do not take into account those cases of GCA with a negative biopsy result [66].

Color duplex ultrasonography has been proposed as a harmless, noninvasive and readily available alternative to temporal artery biopsy for diagnostic purposes [3, 67]. With the demonstration of arterial wall edema (known as the “halo sign”) it may reach a specificity above 90% [3]. However, sensitivity tends to be lower, it is an operator-dependent technique, and specificity may be decreased in patients with atherosclerotic disease [68]. Ultrasonography must therefore be performed by personnel with expertise in the field, in order to avoid false positive and false negative results. Although it could also be an aid to localize the most affected temporal arterial segment for histologic assessment, some studies have not shown superiority of this strategy compared with non-US-guided biopsy [69].

Cranial arterial wall thickening can also be disclosed by means of contrast-enhanced high-resolution MRI, with sensitivities near 90%, but its usefulness decreases when patients have received treatment for more than 5 days and it is a more expensive and less available technique [70–72]. With respect to this, the central bright spot sign (optic nerve head enhancement on MRI) has been proposed as a useful tool to distinguish acute stage GCA from other nonarteritic anterior ischemic optic neuropathies [73].

The use FDG-PET/CT in patients with suspected or confirmed GCA has been progressively increasing in recent years. It allows detection of large-vessel involvement prior or after diagnosis has been made [3, 48, 74] and has special value when there is suspicion of isolated extra-cranial disease [58]. Nevertheless, it is an expensive and not universally available technique and is associated with radiation exposure. Moreover, its sensitivity decreases in patients undergoing glucocorticoid therapy. Some studies comparing FDG-PET/CT and CT-angiography have shown similar sensitivities for both modalities regarding disclosure of large-vessel inflammation, although the former seems to have a higher positive predictive value [75–77]. A cutoff value of > 2.2 mm of aortic wall thickening measured by CT scan has been suggested for diagnostic purposes [78]. The remarkably improved performance of all these imaging modalities and their increasing availability are reflected in the recently published EULAR recommendations, which stated that temporal artery biopsy could be obviated in patients with a strong clinical suspicion and a positive, reliable imaging test [24].

In addition to imaging advances, measurement of autoantibodies against ferritin has also been reported as an alternative diagnostic laboratory biomarker, but its usefulness has not been fully demonstrated yet, and it seems far from everyday clinical practice [79, 80].

GCA is not always a readily suspected condition. Initial diagnosis often relies on primary care physicians and specialists in Geriatrics, so they should be aware of the importance of a prompt detection to avoid serious and irreversible complications due to the delay in treatment. As discussed before, a typical clinical picture is not always present. On the other hand, healthy patients with advanced age may have raised values of erythrocyte sedimentation rate. A survey directed to general practitioners in the UK disclosed that more than 20% of them would rule out GCA if headache was not present among the patient’s symptoms [81]. Other study showed that mean delay from initial symptoms to final diagnosis of GCA was 9 weeks, but this period was almost doubled when there were isolated extra-cranial manifestations [7]. It must be emphasized that GCA is a disease with very heterogeneous signs and symptoms, mimicking many common conditions, and therefore physicians should be familiar with this clinical variability (see Table 1) to be able to raise their clinical suspicion. In this regard, it is essential to educate and train primary care physicians and specialists in Geriatrics and to promote and facilitate communication pathways with other specialists to allow earlier diagnosis [81].

**Table 1:** “Red flags” on history or physical examination that may suggest GCA

Typical cranial disease	
New onset headache (mainly temporal)	
Scalp pain	
Jaw or tongue claudication	
Acute visual deficits	
Temporal artery abnormalities on physical examination	
Anterior ischemic optic neuropathy or central retinal artery occlusion on ophthalmologic examination	
Associated constitutional symptoms	
Associated polymyalgia rheumatica symptoms	
Associated anemia an elevated C reactive protein/erythrocyte sedimentation rate	
Extra-cranial disease	
Ischemic signs and symptoms of extremities, especially in the absence of other cardiovascular risk factors or emboligenic cardiopathy:	
Limb claudication	
Pulse asymmetry	
Arterial pressure asymmetry	
Peripheral arterial bruits	
Distal necrosis or gangrene	
Non-specific manifestations without evidence of infectious o neoplastic disease:	
Fever	
Weight loss	
Fatigue/malaise	
Unexplained anemia	
Polymyalgia rheumatica that relapses or responds poorly to standard glucocorticoid therapy	
Polymyalgia rheumatica with associated ischemic manifestations	
Detection of aneurysm or dissection of aorta and main branches along with raised inflammatory markers	

Table 2 depicts some practical recommendations to improve management, follow-up and prognosis.

**Table 2:** Practical recommendations and learning points in the diagnostic approach and management of GCA

- Former term “temporal arteritis” might be misleading or confounding, as virtually any large or medium-sized artery may be affected.	
- GCA may present with isolated extra-cranial involvement.	
- PMR may be present before, during or after diagnosis of GCA has been established, and vice versa	
- Comprehensive clinical assessment should include palpation of the temporal arteries as well as palpation and auscultation of extracranial vascular territories, including axillary and subclavian arteries, in order to look for any one-sided vascular stenosis. Arterial pressures should also be measured in all four limbs.	
- Temporal artery biopsy is the gold standard for the diagnosis of GCA with cranial manifestations. However, the temporal artery yield decreases significantly in patients presenting with extra-cranial GCA.	
- Temporal artery biopsy is positive in patients with cranial manifestations even several weeks after the onset of glucocorticoids.	
- When there is a very suggestive clinical picture and a positive imaging test, presumptive diagnosis of GCA may be established, thus obviating temporal artery biopsy.	
- Glucocorticoid therapy should be started immediately in patients with a high clinical suspicion of GCA, even before histologic confirmation or imaging tests are available.	
- When ophthalmologic symptoms are present, high doses of glucocorticoids are recommended.	
- After diagnosis of GCA has been established, it may be advisable to use CT-angiography or FDG-PET/CT for screening of large-vessel involvement.	
- Large-vessel complications (aneurysm and dissection) may develop several years after initial diagnosis of GCA, therefore long-term close follow-up is advised.	

## Conclusions

GCA is an inflammatory disease with very heterogeneous and often nonspecific clinical manifestations that can lead to serious and irreversible complications, being visual loss, acute arterial ischemia and acute aortic syndromes the most feared of them. This heterogeneity can lead to a low index of suspicion from attending physicians. It is crucial to establish an early diagnosis and treatment to reduce the risk of developing such complications. It is also important to carry out a close follow-up, since large vessel involvement may occur years after initial recognition of the disease. Newer imaging techniques such as FDG-PET/CT, MRI or CT-angiography can be an aid for diagnosis and also for follow-up of patients.

## Data Availability

Not applicable.

## Abbreviations

CT: Computed tomography
FDG-PET: Fluorodeoxyglucose positron emission tomography
GCA: Giant-cell arteritis
MRI: Magnetic resonance imaging
PMR: Polymyalgia rheumatica
